# IMPPAT: A curated database of Indian Medicinal Plants, Phytochemistry And Therapeutics

**DOI:** 10.1038/s41598-018-22631-z

**Published:** 2018-03-12

**Authors:** Karthikeyan Mohanraj, Bagavathy Shanmugam Karthikeyan, R. P. Vivek-Ananth, R. P. Bharath Chand, S. R. Aparna, Pattulingam Mangalapandi, Areejit Samal

**Affiliations:** 1 The Institute of Mathematical Sciences (IMSc), Homi Bhabha National Institute (HBNI), Chennai, 600113 India; 2 Stella Maris College, Chennai, 600086 India

## Abstract

Phytochemicals of medicinal plants encompass a diverse chemical space for drug discovery. India is rich with a flora of indigenous medicinal plants that have been used for centuries in traditional Indian medicine to treat human maladies. A comprehensive online database on the phytochemistry of Indian medicinal plants will enable computational approaches towards natural product based drug discovery. In this direction, we present, IMPPAT, a manually curated database of 1742 Indian Medicinal Plants, 9596 Phytochemicals, And 1124 Therapeutic uses spanning 27074 plant-phytochemical associations and 11514 plant-therapeutic associations. Notably, the curation effort led to a non-redundant *in silico* library of 9596 phytochemicals with standard chemical identifiers and structure information. Using cheminformatic approaches, we have computed the physicochemical, ADMET (absorption, distribution, metabolism, excretion, toxicity) and drug-likeliness properties of the IMPPAT phytochemicals. We show that the stereochemical complexity and shape complexity of IMPPAT phytochemicals differ from libraries of commercial compounds or diversity-oriented synthesis compounds while being similar to other libraries of natural products. Within IMPPAT, we have filtered a subset of 960 potential druggable phytochemicals, of which majority have no significant similarity to existing FDA approved drugs, and thus, rendering them as good candidates for prospective drugs. IMPPAT database is openly accessible at: https://cb.imsc.res.in/imppat.

## Introduction

Natural products continue to play a significant role in pharmaceutical industry^1–4^ as new sources of drugs. However, recently there has been a decline in the number of marketable drugs derived from natural sources^3,4^. Furthermore, the majority of these drugs fall into already known structural scaffolds as due importance has not been given to unexplored sources of natural products for drug discovery^4^. As a result, lately, there has been significant interest in applying interdisciplinary approaches^5^ to expand the novel chemical scaffold libraries for drug discovery.

India is well known for its practice of traditional medicine and ethnopharmacology^6^. It is noteworthy that traditional Indian medicinal formulations are multi-component mixtures whose therapeutic use is based on empirical knowledge rather than a mechanistic understanding of the active ingredients in the mixture^6^. Until recently, knowledge of traditional Indian medicine including important medicinal plants and their formulations were buried within books such as Indian Materia Medica^7^ and Ayurveda Materia Medica^8^. The nondigital nature of this information limited their effective use towards new drug discovery^5^. Hence, digitization of this knowledge into a comprehensive database on Indian medicinal plants, phytochemistry and ethnopharmacology will enable researchers to apply computational approaches towards drug discovery.

Availability of a curated database of information on plants, their associated natural products and a repository of their chemical structures, can help in *in silico* drug discovery. In this direction, there has been significant recent progress in the development of databases^9–17^ on natural products with a focus on phytochemistry of edible and herbaceous plants. Examples of such databases include CVDHD^12^, KNAPSACK^13^, Nutrichem^9,10^, Phytochemica^11^, TCMID^15^, TCM@Taiwan^14^ and TCM-Mesh^16^ which can facilitate virtual screening of prospective drug compounds or aid in the investigation of plant-disease associations. However, from the perspective of traditional Indian medicine, there have been relatively few efforts to build online databases that include Indian medicinal plants, their phytochemicals and therapeutic uses. Previously, Polur *et al*.^18^ compiled information on 295 ayurvedic Indian medicinal plants, their 1829 phytochemicals and therapeutic uses. Subsequently, Polur *et al*.^18^ studied the structural similarity between their library of 1829 phytochemicals and drugs in the DrugBank^19^ database to predict pharmacologically active natural compounds. Recently, the Phytochemica^11^ database gathered information on 5 Indian medicinal plants and their 963 phytochemicals. In addition, Phytochemica^11^ provided chemical structures and pharmacological properties of the phytochemicals within their database. Other efforts to build online databases for traditional Indian medicine has largely been limited to cataloguing medicinal plants and their therapeutic uses rather than capturing the phytochemicals that are vital for drug discovery. On the other hand, in contrast to the above mentioned online databases, more comprehensive databases are available for Chinese medicinal plants^14–16^. For example, TCM-MeSH^16^ is an online database for traditional Chinese medicine which captures phytochemical compositions and therapeutic uses for more than 6000 Chinese medicinal plants.

We therefore have built a manually curated database, IMPPAT, containing 1742 Indian Medicinal Plants, 9596 Phytochemicals, And 1124 T herapeutic uses. In addition, the IMPPAT database has linked Indian medicinal plants to 974 openly accessible traditional Indian medicinal formulations. Importantly, our curation efforts have led to a non-redundant *in silico* chemical library of 9596 phytochemicals with two-dimensional (2D) and three-dimensional (3D) chemical structures. For the 9596 phytochemicals in our database, we have computed physicochemical properties and predicted Absorption, distribution, metabolism, excretion and toxicity (ADMET) properties using cheminformatic tools^20–22^. We then employed cheminformatic approaches to evaluate the drug-likeliness of the phytochemicals in our *in silico* chemical library using multiple scoring schemes such as Lipinski’s rule of five (RO5)^23^, Oral PhysChem Score (Traffic Lights)^24^, GlaxoSmithKline’s (GSK’s) 4/400^25^, Pfizer’s 3/75^26^, Veber rule^27^ and Egan rule^28^. We found a subset of 960 phytochemicals of Indian medicinal plants that are potentially druggable in our chemical library of 9596 phytochemicals based on multiple scoring schemes. We also provide predicted interactions between phytochemicals in our database and human target proteins from STITCH^29^ database. Table 1 provides a comparison of the IMPPAT database with previous efforts by Polur *et al*.^18^ and Phytochemica^11^ to build dedicated digital resource on phytochemical composition of Indian medicinal plants. In summary, IMPPAT is the largest database on phytochemicals of Indian medicinal plants to date, and this resource is a culmination of our efforts to digitize the wealth of information contained within traditional Indian medicine. IMPPAT provides an integrated platform to apply cheminformatic^30^ approaches to accelerate natural product based drug discovery. IMPPAT is openly accessible at: https://cb.imsc.res.in/imppat.

**Table 1:** Comparison of IMPPAT with earlier databases on phytochemical composition of Indian medicinal plants.

Database	IMPPAT	Phytochemica^11^	Polur *et al*.^18^
**Basic statistics**			
Number of Indian medicinal plants	1742	5	295
Number of phytochemicals	9596	963	1829
**Type of associations**			
Plant-phytochemical associations	Yes	Yes	Yes
Plant-therapeutic use associations	Yes	No	Yes
Plant-medicinal formulation associations	Yes	No	No
Phytochemical-human target protein associations	Yes	No	Yes
Plant part-phytochemical associations	No	Yes	No
**Additional Features**			
Web interface	Yes	Yes	No
Availability of 2D structure of phytochemicals	Yes	No	No
Availability of 3D structure of phytochemicals	Yes	Yes	No
Downloadable structure file formats	MOL, MOL2, SDF, PDB & PDBQT	MOL2	No
Chemical classification	Yes	Yes	No
Physicochemical properties	Yes	Yes	No
ADMET properties	Yes	Yes	No
Druggability properties	Yes	No	No
Cytoscape network visualization of associations	Yes	No	No
Filter phytochemicals based on physicochemical properties	Yes	Yes	No
Filter phytochemicals based on druggability properties	Yes	No	No
Chemical similarity search within database	Yes	No	No

## Methods

### Curated list of Indian medicinal plants

**Figure 1:**
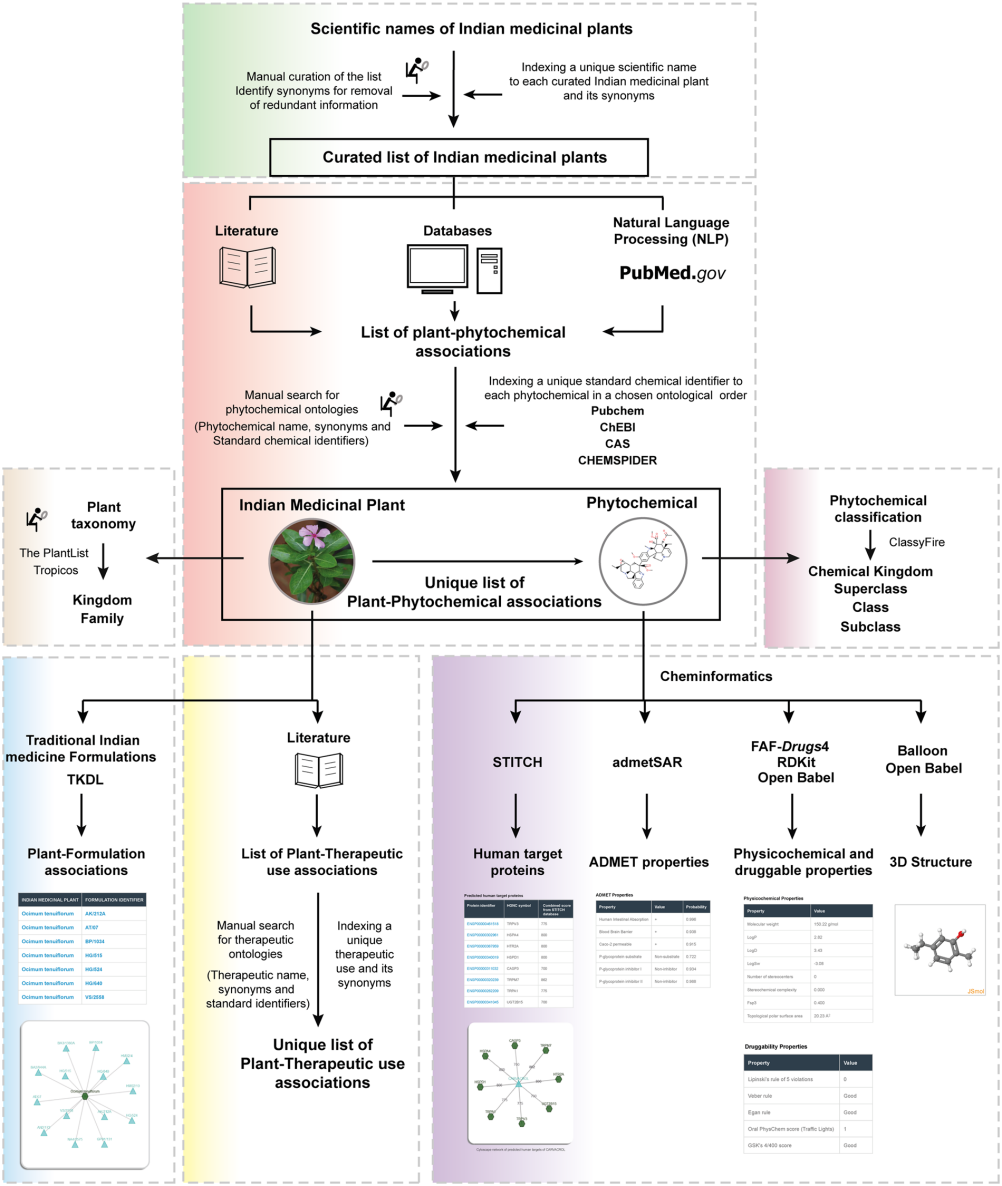
Schematic overview of the IMPPAT database construction pipeline. Briefly, we first compiled a comprehensive list of Indian medicinal plants from various sources. We next mined specialized books on Indian traditional medicine, existing databases and PubMed abstracts of journal articles to gather information on phytochemicals of Indian medicinal plants. We then manually annotated, curated and indexed names of identified phytochemicals with standard identifiers to build a non-redundant library of phytochemicals. This manual curation effort led to a unique list of plant-phytochemical associations. We also classified the Indian medicinal plants into taxonomic families and phytochemicals into chemical classes. Subsequently, we gathered ethnopharmacological information from books on traditional Indian medicine to build a unique list of plant-therapeutic use associations. We also extracted publicly accessible information on traditional medicine formulations from TKDL database to build a list of plant-formulation associations. Lastly, we have used cheminformatic tools to obtain the 3D structures, physicochemical properties, druggability scores, predicted ADMET properties and predicted target human proteins of phytochemicals.

In the preliminary phase of the database construction (Fig. 1), we compiled a comprehensive list of more than 5000 Indian medicinal plants based on information contained in the Indian medicinal plants database (http://www.medicinalplants.in/) of the Foundation for Revitalisation of Local Health Traditions (FRLHT), Bengaluru. In addition to the comprehensive list from FRLHT, the AYUSH priority list was compiled from two sources, namely, the list prepared by the National Mission on Medicinal Plants of Ministry of AYUSH, Government of India which is available at http://ayush.gov.in/sites/default/files/IV.pdf, and the list jointly prepared by the National Medicinal Plants Board (NMPB), Directorate of Medicinal and Aromatic Plants Research (DMAPR), Department of Agriculture and Cooperation, and Central Institute of Medicinal and Aromatic Plants (CIMAP), Government of India which is available at http://pib.nic.in/newsite/PrintRelease.aspx?relid = 67277. We remark that the AYUSH priority list was prepared based on several criteria including the medicinal use, conservation status and herbal industry demand of Indian medicinal plants. Due to the usage of multiple synonyms for Indian medicinal plants across different sources, the common names of plants were manually mapped to their scientific species names using The Plant List database^31^ (http://www.theplantlist.org/), and the compiled list was manually curated to remove redundancies. Furthermore, the Indian medicinal plants in our database were manually classified into their respective taxonomic families within the kingdom plantae using The Plant List database^31^ and Tropicos database (http://www.tropicos.org/). We have also linked the Indian medicinal plants in IMPPAT database to their corresponding page in The Plant List database, Tropicos database and the FRLHT digital herbarium (http://envis.frlht.org).

### Phytochemical composition of Indian medicinal plants

After compiling a comprehensive list of more than 5000 Indian medicinal plants, we mined literature to gather information on their phytochemicals (Fig. 1). In the first stage of data mining, we focussed on specialized traditional Indian medicine books^32–41^. From these books^32–41^, we gathered phytochemical composition for more than 1600 Indian medicinal plants. In the second stage, we gathered information from published databases of Indian medicinal plants. Phytochemica^11^ database contains information on 963 phytochemicals of 5 Indian medicinal plants. Another database described in Polur *et al*.^18^ had compiled information on 1829 phytochemicals of 295 ayurvedic Indian medicinal plants^18^. While this list is no longer publicly available, the Nutrichem^9,10^ database on phytochemical composition and therapeutic uses of plant-based food products has incorporated the information compiled by Polur *et al*.^18^. From the Phytochemica^11^ and Nutrichem^9,10^ databases, we gathered information on the phytochemical composition of more than 400 Indian medicinal plants. Note that our comprehensive list covers a wide spectrum of Indian medicinal plants which includes apart from Ayurveda, other systems of traditional Indian medicine such as Siddha and Unani. In the third stage of data mining for phytochemical composition, we performed text mining of abstracts from published research articles in PubMed^42^ using natural language processing (NLP)^43^. Using in-house Python scripts and a dataset of known plant-phytochemical associations, we identified keywords in PubMed abstracts which imply plant-phytochemical associations (Supplementary Table S1). We then used the selected keywords listed in Supplementary Table S1 to mine PubMed abstracts to identify and incorporate additional references for plant-phytochemical associations in our database. In total, our database captures the phytochemical composition of 1742 Indian medicinal plants (Supplementary Table S2). The literature references for plant-phytochemical associations are listed in our database in the form of ISBN or DOI identifiers for books and PubMed identifiers (PMIDs) for journal articles.

We would like to mention a potential bias in the list of plant-phytochemical associations compiled from scientific literature. Our database most-likely contains high-quality yet incomplete information on phytochemical composition of Indian medicinal plants. That is, phytochemicals listed are most-likely produced by the corresponding Indian medicinal plant but other phytochemicals not listed in our database cannot be ruled out from being also produced by the same plant due to possible lack of scientific literature. Moreover, the scientific literature will most probably have more information on phytochemical composition of well-studied or sequenced Indian medicinal plants such as *Catharanthus roseus*. Thus, future updates of this database will be needed to capture additional information on phytochemical composition of Indian medicinal plants. Nevertheless, one can argue that for the discovery of novel molecules it is more important to know the list of phytochemicals produced by an herb rather than the list of phytochemicals not produced by an herb.

### Annotation, curation and filtering of identified phytochemicals

An overarching goal of this work is to create a platform for exploring the chemistry of the phytochemicals of Indian medicinal plants. Evaluation of the phytochemicals of Indian medicinal plants for their druggability or drug-likeliness will facilitate the identification of molecules for drug discovery. We would like to emphasize that synonymous chemical names are pervasive across the literature on traditional Indian medicine which were mined to construct this database. In order to remove redundancy, we manually annotated the common names of phytochemicals of Indian medicinal plants compiled from literature sources with documented synonyms and standard chemical identifiers (Fig. 1) from Pubchem^44^, CHEBI^45^, CAS (https://www.cas.org/), CHEMSPIDER^46^, KNAPSACK^47^, CHEMFACES (http://www.chemfaces.com), FOODB (http://foodb.ca/), NIST Chemistry webbook^48^ and Human Metabolome database (HMDB)^49^. While assigning standard identifiers to phytochemicals in our database, we have chosen the following priority order: Pubchem^44^, CHEBI^45^, CAS, CHEMSPIDER^46^, KNAPSACK^47^, CHEMFACES, FOODB, NIST Chemistry webbook^48^ and HMDB^49^. We highlight that this extensive manual curation effort led to the mapping of more than 15000 common names of phytochemicals used across literature sources to a unique set of 9596 standard chemical identifiers. Phytochemicals which could not be mapped to standard chemical identifiers were excluded from our finalized database. Our choice to include only phytochemicals with standard identifiers and structure information was dictated by our goal to investigate the chemistry and druggability of phytochemicals of Indian medicinal plants. We remark that the 2D structure information for the unique set of 9596 IMPPAT phytochemicals was obtained using the standard chemical identifiers from the respective databases. We have also determined the chemical classification of the IMPPAT phytochemicals using ClassyFire^50^ (http://classyfire.wishartlab.com/). ClassyFire^50^ gives a hierarchical classification for each chemical compound into kingdom (organic or inorganic), followed by super-class, followed by class, followed by sub-class. Note that ClassyFire classifies organic compounds into 26 super-classes. In a nutshell, this largely manual effort to compile a non-redundant chemical library of 9596 phytochemicals of Indian medicinal plants with standard identifiers and structure information will serve as valuable resource for natural product-based drug discovery in future. Moreover, the use of standard chemical identifiers will enable effortless integration of our IMPPAT database with other data sources.

### Therapeutic uses of Indian medicinal plants

Another goal of our database is to compile ethnopharmacological information on Indian medicinal plants. Towards this goal, we manually compiled the medicinal (therapeutic) uses of Indian medicinal plants from books on Indian traditional medicine^32–41,48,51–67^. Apart from books, Polur *et al*.^18^ had previously compiled a list of therapeutic uses for 295 ayurvedic Indian medicinal plants, and this information was extracted from the Nutrichem^9,10^ database. To ensure high quality, we manually curated information on therapeutic uses of Indian medicinal plants and consciously avoided automated text mining to retrieve additional information on plant-therapeutic associations. We remark that our database has manually compiled therapeutic uses of Indian medicinal plants from standard books on traditional Indian medicine which contain accumulated experience-based knowledge on treating human diseases. Furthermore, we manually annotated and standardized the compiled therapeutic uses of Indian medicinal plants from the above sources with identifiers from the Disease Ontology^68^, Online Mendelian Inheritance in Man (OMIM)^69^, Unified Medical Language System (UMLS)^70^ and Medical Subject Headings (MeSH)^71^ databases. To the best of our knowledge, this is the first large-scale attempt to link the ethnopharmacological information on Indian medicinal plants with standardized vocabulary in modern medicine. Note that databases of gene-disease associations^72^ and disease-symptom associations^73^ usually provide disease information in form of identifiers from OMIM, UMLS and MeSH databases, and in future, information from such databases can be effortlessly integrated into IMPPAT database.

### Traditional formulations of Indian medicinal plants

Traditional knowledge digital library (TKDL) (http://www.tkdl.res.in) is a knowledgebase of traditional Indian medicinal formulations. A traditional medicinal formulation is often a multi-component mixture derived from plant, animal and other sources which is used for treating disease based on specific indication. For example, Thinavu Sori Soolaiku Ennai (TKDL Identifier: HM02/36) is a medicinal formulation in traditional Indian system of medicine, Siddha, which is used to treat allergic rashes, and this formulation mainly consists of extracts of medicinal plants, *Plumbago zeylanica*, *Sesamum orientale* (also called *Sesamum indicum*) and *Cuminum cyminum*. According to TKDL, there are more than 250000 formulations of Ayurveda, Siddha and Unani of which 1200 representative formulations are openly accessible via their database. To exhibit the broader utility of our database to phytopharmacology, we have also compiled and curated the subset of 1200 openly accessible formulations in TKDL which contain at least one of the 1742 Indian medicinal plants in our database. This process led to associations between 321 Indian medicinal plants in our database and 974 traditional Indian medicinal formulations which are openly accessible through TKDL database (Fig. 1). We emphasize that our database has only incorporated open digital information on traditional Indian medicinal formulations from TKDL database. However, we are aware of the vast literature^7,8,74^ on traditional Indian medicinal formulations, especially in books, and in the future, a significant effort will be needed to digitize and integrate such information into our database.

### 3D structure of phytochemicals

We have generated lowest energy 3D conformational structure of IMPPAT phytochemicals using Balloon^75^ (http://users.abo.fi/mivainio/balloon/) and Open Babel^76^ (http://openbabel.org/wiki/Main_Page). Balloon generates 3D conformers of input 2D structures from scratch and optimizes them using Merck Molecular Force Field (MMFF94). The lowest energy 3D conformer was selected from 20 generated conformations for a given phytochemical. Of the 9596 IMPPAT phytochemicals, Balloon successfully generated 3D structures for 8021 phytochemicals. For the remaining 1575 phytochemicals, the lowest energy 3D conformer was generated using Open Babel with MMFF94 force field. We remark that our preferred choice of Balloon to generate lowest energy 3D structures of IMPPAT phytochemicals was motivated by similar choice made by two other databases of 3D structures of natural products, namely, KNApSAcK-3D^47^ and TIPdb-3D^77^.

### Physicochemical properties of phytochemicals

We used FAF-Drugs4 webserver^20^ and RDKit^21^ to compute the following physicochemical properties of the IMPPAT phytochemicals: molecular weight, octanol-water partition coefficient (logP), logP at physiological pH of 7.4 (logD), logarithm of water solubility (logSw), number of stereocenters, stereochemical complexity^78^ which is the fraction of carbon atoms which are stereogenic, Fsp^3^ which is the fraction of carbon atoms that are sp^3^ hybridized^79^, topological polar surface area (TPSA), charge of the compound, number of hydrogen bond donors and acceptors, number of smallest set of smallest rings (SSSR) which is the number of smallest ring building blocks required for forming other ring systems, size of the biggest system ring which is the number of atoms present in the biggest ring system, number of rotatable and rigid bonds, number of charged groups, total charge of the compound, number of carbon, hetero- and heavy atoms, and ratio between the number of non-carbon atoms and the number of carbon atoms.

### ADMET properties of phytochemicals

Absorption, distribution, metabolism, excretion and toxicity (ADMET) properties have been implicated as one of the reasons for high attrition rate of candidates from drug development pipeline. Thus, we used admetSAR^22^ webserver to predict the ADMET properties of the phytochemicals. The predicted properties which influence absorption include Human Intestinal Absorption (HIA)^80^, Blood Brain Barrier (BBB) permeability^80^, Caco-2 permeability^81^ and likeliness of being P-glycoprotein substrate^82^. The predicted properties which affect phytochemical metabolism include the ability to inhibit several CYP450 enzymes or likeliness of being a substrate to CYP450 enzymes^83–85^. Lastly, toxicity predictions are based on computational models for Ames test for mutagenicity^86^, carcinogenicity, biodegradability^87^, rat acute toxicity^88^ and hERG inhibition^89,90^. Note that our choice of admetSAR^22^ was motivated by the same choice made by DrugBank^19^ database (https://www.drugbank.ca/) which is the widely-used repository of approved and experimental drugs.

### Druggability scores of phytochemicals

We used FAF-Drugs4 webserver^20^ to test the druggability of the phytochemicals based on multiple scoring schemes, namely, Lipinski’s rule of five (RO5)^23^, Oral PhysChem^24^ score (Traffic Lights), GlaxoSmithKline’s (GSK’s) 4/400^25^, Pfizer’s 3/75^26^, Veber rule^27^ and Egan rule^28^. Lipinski’s RO5^23^ is a classical rule of thumb to filter druggable small molecules based on four physiochemical properties. RO5 considers a small molecule to be druggable if it has ≤5 hydrogen bond donors, ≤10 hydrogen bond acceptors, molecular weight <500 Daltons and logP ≤5. If a small molecule violates none of the above rules it is assigned a RO5 value of 0, and on the other extreme if it violates all the above rules it is assigned a RO5 value of 4. OralPhysChem^24^ score is another method for filtering druggable small molecules which is based on five physiochemical properties, namely, aqueous solubility, logP, corrected molecular weight for presence of halogen atoms, TPSA and number of rotatable bonds. OralPhysChem score ranges from 0 to 10 whereby 0 signifies high druggability while 10 signifies low druggability of the small molecule. GSK’s 4/400^25^ is another filter based on a number of ADMET assays carried out in GSK. Briefly, a small molecule is considered more druggable and labelled ‘Good’ by GSK’s 4/400 score if it has both molecular weight <400 Daltons and logP <4 while it is considered less druggable and labelled ‘Bad’ if at least one of the rules is not satisfied. Pfizer’s 3/75^26^ rule is used to filter small molecules which are more prone to be toxic, and hence, less likely to be druggable. Pfizer’s 3/75 rule considers small molecules with logP <3 and TPSA > 75 Å^2^ to be ‘Good’ as they are likely to be less toxic, and hence, more likely to be druggable, and small molecules which do not satisfy one of the two rules are labelled as ‘Warning’, and small molecules which violate both rules are labelled as ‘Bad’. Veber rule^27^ considers small molecules to have good oral bioavailability if they satisfy number of rotatable bonds ≤10 and TPSA ≤140 Å^2^, and small molecules which fail these criteria are considered to have low bioavailability. Similarly, Egan rule^28^ considers small molecules to have good oral bioavailability if they satisfy −1.0 ≤logP ≤5.8 and TPSA ≤130 Å^2^, and small molecules which fail these criteria are considered to have low bioavailability. We filtered phytochemicals with no RO5 violation, net Traffic Lights value of zero and satisfying GSK’s 4/400, Pfizer’s 3/75, Veber rule and Egan rule as *druggable*. We further computed the weighted quantitative estimate of drug-likeness (QEDw)^91^ score using FAF-QED webserver^20^ for the filtered list of druggable phytochemicals within IMPPAT and TCM-Mesh^16^. QEDw is a druggability score for small molecules proposed by Bickerton *et al*.^91^ which is the weighted geometric mean of molecular weight, logP, number of hydrogen bond donors, number of hydrogen bond acceptors, TPSA, number of rotatable bonds, number of aromatic rings and number of structural alerts. Note that QEDw is a continuous score between 0 and 1 where 0 signifies low druggability and 1 signifies high druggability.

### Predicted human target proteins of phytochemicals

We have extracted the predicted human target proteins of IMPPAT phytochemicals from STITCH^29^ database (http://stitch1.embl.de/). Note that STITCH^29^ database is the largest resource on predicted interactions between chemicals and their target proteins. From the STITCH^29^ database, we have extracted and reported only high confidence interactions between phytochemicals and target human proteins that have a combined STITCH score ≥700. Note that our choice of STITCH database to predict interactions between IMPPAT phytochemicals and target human proteins was based on similar choice made by the traditional Chinese medicine database TCM-Mesh^16^.

### Small molecule collections of commercial compounds, diverse compounds, natural products and phytochemicals from Chinese medicinal plants

We have compared the physicochemical properties of 9596 IMPPAT phytochemicals from Indian medicinal plants with other collections of small molecules. Clemons *et al*.^78^ have compiled small molecule collections from three different sources, namely, commercial compounds (CC), diversity-oriented synthesis compounds (DC’) and natural products (NP). CC contains 6152 representative small molecules from commercial sources. DC’ contains 5963 small molecules synthesized by academic community using methods like diversity-oriented synthesis. NP contains 2477 small molecules from natural products. We remark that 11, 3 and 147 small molecules in CC, DC’ and NP collections, respectively, are also contained in the set of 9596 IMPPAT phytochemicals. Note that the computation of physicochemical properties failed for 3 small molecules in CC and 3 small molecules in DC’, and we omitted these small molecules from subsequent analysis.

In addition, we have also extracted the set of 10140 phytochemicals produced by 6235 Chinese medicinal plants or herbs from TCM-Mesh^16^ (http://mesh.tcm.microbioinformatics.org/) database. TCM-Mesh has compiled information on phytochemicals of Chinese herbs from two other extensive databases on traditional Chinese medicine, TCMID^15^ (http://www.megabionet.org/tcmid/) and TCM@Taiwan^14^ (http://tcm.cmu.edu.tw/). Note that TCM-Mesh contains a large set of 383840 chemical compounds but only a subset of 10140 phytochemicals are ingredients of 6235 Chinese herbs in the database.

### Similarity of phytochemicals

Tanimoto coefficient (Tc)^92^ is a widely used measure to compute structural similarity between chemicals^93^. To evaluate the structural similarity of chemicals within our database to known drugs using Tc, we employed two molecular fingerprints: (a) Extended Circular Fingerprints (ECFP4)^94^ applying Morgan algorithm^95^ with radius value of 2 as implemented in RDKit^21^, and (b) MACCS keys based fingerprint. We employed the open source package, RDKit^21^, to compute molecular fingerprints and Tc between pairs of chemical structures. To identify structural similarity between chemicals, a stringent cut-off of Tc ≥0.5 was used while employing ECFP4 and a cut-off of Tc ≥0.85 was used while employing MACCS keys. Our selection of Tc cut-offs for ECFP4 and MACCS keys based computations was motivated by the recent work of Jasial *et al*.^96^.

We obtained a list of 2069 FDA approved drugs from DrugBank^19^ database and computed their structural similarity with druggable IMPPAT phytochemicals using both ECFP4 and MACCS keys based molecular fingerprints. Note that ECFP4 molecular fingerprints were used to create the chemical similarity network of the druggable phytochemicals with QEDw score ≥0.9. Besides quantifying the structural similarity based on the Tc of phytochemicals, we have employed principal component analysis (PCA) to explore possible relationships between druggable phytochemicals with QEDw score ≥0.9 based on their physicochemical properties.

### Database management and network visualization

**Figure 2:**
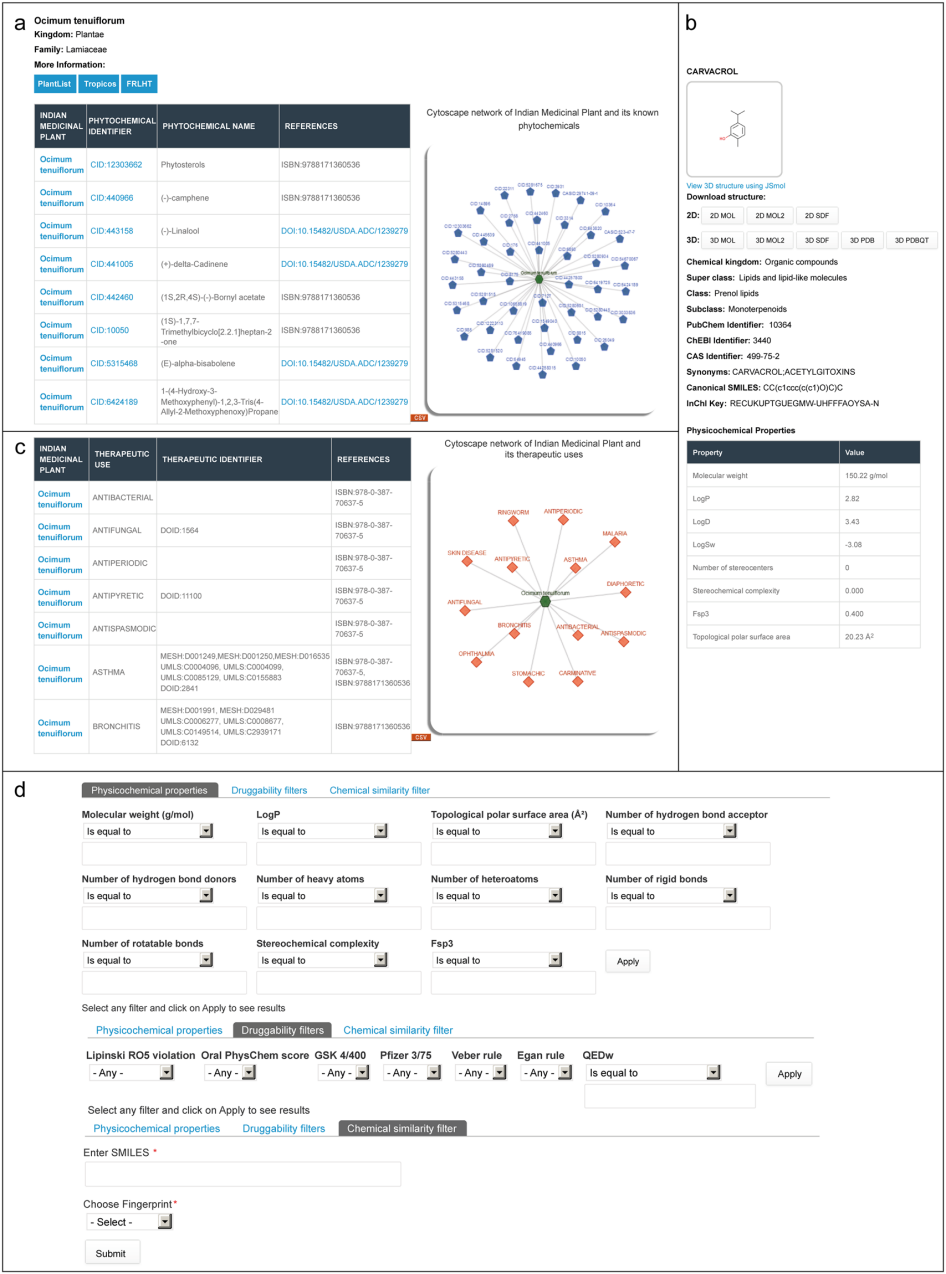
Web-interface of the IMPPAT database. (**a**) Snapshot of the result of a standard query for phytochemicals of an Indian medicinal plant. In this example, we show the plant-phytochemical association for *Ocimum tenuiflorum*, commonly known as Tulsi, from IMPPAT database. (**b**) Snapshot of the dedicated page containing detailed information on 2D and 3D chemical structure, physicochemical properties, druggability scores, predicted ADMET properties and predicted target human proteins for a chosen phytochemical. From the dedicated page for each phytochemical, users can download the 2D and 3D structure of the phytochemical in the form of a SDF or MOL or MOL2 or PDB or PDBQT file. (**c**) Snapshot of the result of a standard query for therapeutic uses of an Indian medicinal plant. In this example, we show the therapeutic uses of *Ocimum tenuiflorum* from IMPPAT database. (**d**) Snapshot of the advanced search options which enable users to filter phytochemicals based on their physiochemical properties or druggability scores or chemical similarity with a query compound.

To construct this database, the compiled and curated data was integrated using MySQL (https://www.mysql.com/), a relational database management system which serves as a back-end for our resource. The web interface for the database was built using Drupal (https://www.drupal.org/), a PHP-based content management system hosted on Apache server with the MySQL database in the back-end. Users can browse or query our database using the scientific names of Indian medicinal plants, standard identifiers for phytochemicals, or associated therapeutic uses (Fig. 2). Further we have integrated the Cytoscape.js application^97^ (http://js.cytoscape.org/) into our web interface which enables visualization of plant-phytochemical associations, plant-therapeutic associations, and plant-formulation associations in the form of a network. The Cytoscape network visualization displays different types of nodes such as plant, phytochemical, therapeutic use and traditional medicinal formulations in different shapes and colours. Finally, the association network can be downloaded as a tab-separated list using the available export option in our database (Fig. 2).

### Data availability

The datasets generated and analysed in this study are openly accessible at: https://cb.imsc.res.in/imppat.

## Results

### Web-interface of the database

The IMPPAT database captures information on three types of associations for Indian medicinal plants: phytochemical composition, therapeutic uses, and traditional medicinal formulations (Fig. 1). The web-interface of the database enables users to query for each of these associations using (a) scientific names of plants, (b) standard chemical identifiers of phytochemicals, (c) therapeutic uses, or (d) formulation identifiers (Fig. 2). The web-interface displays the result of user queries for these associations in two ways: (a) A table of associations with references to literature sources, and (b) A network visualization of the associations which is powered by Cytoscape.js^97^ (Fig. 2). In addition, users can also download the result of their queries for different associations of medicinal plants as a tab-separated list using the available export option in the web-interface. In the results page of queries for plant-phytochemical associations, users can click each phytochemical name or identifier to navigate to a separate page containing detailed information such as chemical structure, alternate chemical names or identifiers, computed physicochemical properties, computed druggability scores, predicted ADMET properties, predicted human target proteins and the option to download the 2D or 3D chemical structure file in several formats (Fig. 2; Methods). Queries for plant-therapeutic associations leads to a page where users can also obtain the disease ontology identifiers corresponding to therapeutic uses (Fig. 2; Methods). In the results page of queries for plant-formulation associations, users can click the medicinal formulation identifiers to navigate to the corresponding page in the TKDL database. Moreover, in the advanced search page of IMPPAT database (Fig. 2), users can filter phytochemicals based on physicochemical properties (e.g., molecular weight, number of hydrogen bond acceptors), or filter phytochemicals satisfying various druggability scores (e.g. RO5, Traffic Lights), or search for phytochemicals similar to query chemical compound. To run the similarity filter, users will have to provide the query compound in the form of Canonical SMILES and choose a molecular fingerprint (ECFP4 or MACCS keys) to compute Tc between the query compound and IMPPAT phytochemicals. The chemical similarity filter will list top 10 IMPPAT phytochemicals which are similar to the input query compound based on Tc.

### Network of plant-phytochemical associations, plant-therapeutic use associations, and plant-traditional medicinal formulation associations

**Figure 3:**
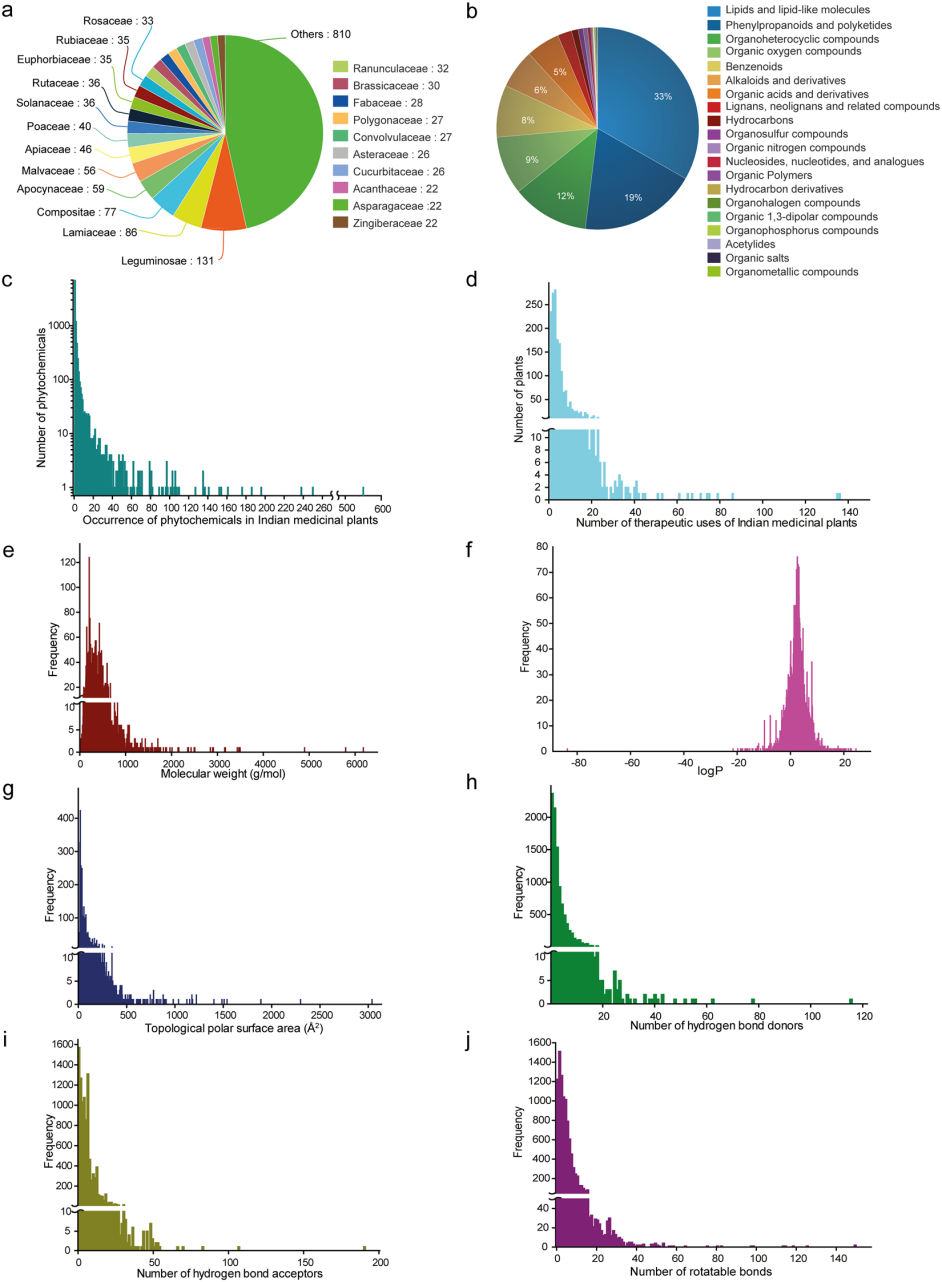
Basic statistics for Indian medicinal plants and associated phytochemicals in IMPPAT database. (**a**) Pie chart shows the distribution of the 1742 Indian medicinal plants in IMPPAT database across different taxonomic families. (**b**) Pie chart shows the distribution of the 9596 IMPPAT phytochemicals across different chemical super-classes obtained from ClassyFire^50^. (**c**) Histogram of the number of Indian medicinal plants which produce a given phytochemical in our database. (**d**) Histogram of the number of therapeutic uses per Indian medicinal plant in our database. (**e**–**j**) Histogram of the molecular weight (in g/mol), logP, TPSA (in Å^2^), number of hydrogen bond donors, number of hydrogen bond acceptors and number of rotatable bonds of the phytochemicals in our database.

IMPPAT database contains information on the phytochemical composition and therapeutic uses of 1742 Indian medicinal plants (Supplementary Table S2). The 1742 Indian medicinal plants in our database are distributed across 215 different taxonomic families (Fig. 3a; Methods). Among the 215 taxonomic families, Leguminosae contains the maximum number (131) of Indian medicinal plants in our database (Fig. 3a). Of the 134 Indian medicinal plants in the priority list of Ministry of AYUSH, Government of India, 116 Indian medicinal plants are contained in our database (Supplementary Table S2).

IMPPAT captures information on 27074 plant-phytochemical associations which encompasses 1742 Indian medicinal plants and their 9596 phytochemicals. We used ClassyFire^50^ webserver for chemical classification of the 9596 IMPPAT phytochemicals (Methods). The 9596 IMPPAT phytochemicals are distributed across 24 super-classes, 260 classes and 415 sub-classes of ClassyFire^50^. Among the 24 super-classes, lipids and lipid-like molecules, phenylpropanoids and polyketides, and organoheterocyclic compounds are the top three super-classes with 3190, 1793 and 1184 phytochemicals, respectively (Fig. 3b). Among 260 chemical classes, prenol lipids, organooxygen compounds, and flavonoids are the top three classes with 2005, 868 and 818 phytochemicals, respectively. Among the 1742 Indian medicinal plants in our database, *Catharanthus roseus* has the highest number of phytochemical associations. In Fig. 3c, we show a histogram of the occurrence of phytochemicals across 1742 Indian medicinal plants in our database. From this figure, it is seen that the majority of (8838) phytochemicals are found in less than 5 Indian medicinal plants while only a handful of (3) phytochemicals are found in more than 200 Indian medicinal plants. IMPPAT also captures 48632 predicted interactions between phytochemicals and their human target proteins from STITCH^29^ database which encompasses 1477 IMPPAT phytochemicals and 8128 human proteins (Methods).

IMPPAT also captures information on 11514 plant-therapeutic use associations which encompasses 1742 Indian medicinal plants and 1124 therapeutic uses. In Fig. 3d, we show a histogram of the number of therapeutic uses per Indian medicinal plant in our database. From this figure, it is seen that a majority of 1409 Indian medicinal plants have less than 10 documented therapeutic uses while a small fraction of 90 Indian medicinal plants have more than 20 therapeutic uses in our database. Among the 1742 Indian medicinal plants in our database, *Ginkgo biloba* (136), *Panax ginseng* (135) and *Allium sativum* (86) have the largest number of documented therapeutic uses. Lastly, IMPPAT also captures information on 5069 plant-formulation associations which encompasses 321 Indian medicinal plants in our database and 974 traditional Indian medicinal formulations which are openly accessible from the TKDL database (Methods).

### Comparative analysis of the physicochemical properties of IMPPAT phytochemicals with other small molecule collections

**Figure 4:**
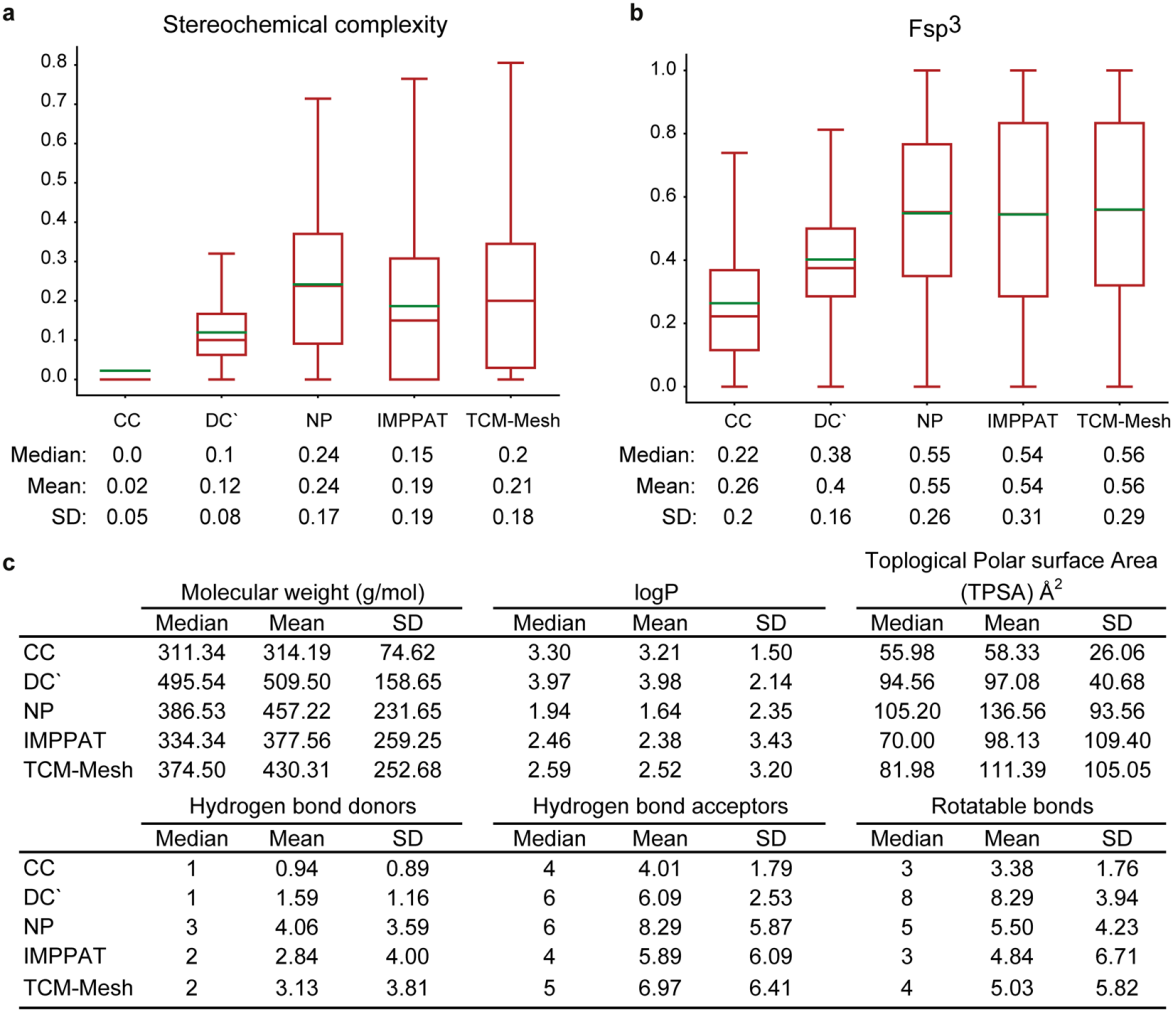
Comparison of the physicochemical properties of IMPPAT phytochemicals with other small molecule collections. (**a**) Box plot shows the distribution of the stereochemical complexity of the small molecule collections CC, DC’, NP, IMPPAT phytochemicals and TCM-Mesh phytochemicals. The median, mean and standard deviation (SD) of the stereochemical complexity for each small molecule collection is shown below the box plot. (**b**) Box plot shows the distribution of the Fsp^3^ for the small molecule collections CC, DC’, NP, IMPPAT phytochemicals and TCM-Mesh phytochemicals. The median, mean and SD of the Fsp^3^ for each small molecule collection is shown below the box plot. Note the lower end of the box shows the first quartile, upper end of the box shows the third quartile, brown line shows the median and green line shows the mean of the distribution of stereochemical complexity or Fsp^3^ in the two box plots. (**c**) Median, mean and SD of six physicochemical properties, namely, molecular weight, logP, TPSA, number of hydrogen bond donors, number of hydrogen bond acceptors and number of rotatable bonds for the small molecule collections CC, DC’, NP, IMPPAT phytochemicals and TCM-Mesh phytochemicals.

We have computed several physicochemical properties for the 9596 IMPPAT phytochemicals (Methods). Figure 3e–j shows the distribution and the table in Fig. 4c gives the mean and median of the distribution for six physicochemical properties, namely, molecular weight, logP, TPSA, number of hydrogen bond donors, number of hydrogen bond acceptors and number of rotatable bonds for the 9596 IMPPAT phytochemicals. Moreover, we have predicted several ADMET properties for the 9596 IMPPAT phytochemicals (Methods). For example, HIA model predicts 89% of IMPPAT phytochemicals have good intestinal absorption and carcinogenicity model predicts 94% of IMPPAT phytochemicals are non-carcinogenic.

Small molecules which are specific protein binders in screening assays are more favourable candidates for drug discovery pipeline than promiscuous binders which might interact with many proteins in a screening assay. Clemons *et al*.^78^ have correlated two simple size-independent metrics, namely, stereochemical complexity and shape complexity (Fsp^3^)^79^ with the binding specificity of representative compound collections, CC, DC’ and NP (Methods). Clemons *et al*.^78^ found that DC’ and NP collections have more stereochemical complexity and shape complexity in comparison to CC collection, and interestingly, small molecules in DC’ and NP collections were shown to be more specific binders with less fraction of promiscuous binders in comparison to small molecules in CC collection. We have compared the distribution of stereochemical complexity and Fsp^3^ across 9596 IMPPAT phytochemicals with CC, DC’ and NP collections from Clemons *et al*.^78^ and 10140 TCM-Mesh^16^ phytochemicals from Chinese medicinal plants (Fig. 4a-b; Methods). Interestingly, we find the mean and median of stereochemical complexity of IMPPAT phytochemicals is higher than CC and DC’ collections while being closer to NP collection (Fig. 4a). Furthermore, the mean and median of stereochemical complexity of IMPPAT phytochemicals was found to be much closer to TCM-Mesh phytochemicals in comparison to DC’, CC or even NP collection (Fig. 4a). We also obtain similar trends for the mean and median of shape complexity (Fsp^3^) of IMPPAT phytochemicals (Fig. 4b). These observations underscore that the IMPPAT phytochemicals are closer to small molecule libraries of natural products or phytochemicals from Chinese medicinal plants in terms of stereochemical complexity and Fsp^3^, and thus, are more likely enriched with specific binders than promiscuous binders.

In a later study, Clemons *et al*.^98^ have also shown that the small molecules in CC, DC’ and NP occupy different regions in the physicochemical space. By considering six physicochemical properties studied by Clemons *et al*.^98^, namely, molecular weight, logP, TPSA, number of hydrogen bond donors, number of hydrogen bond acceptors and number of rotatable bonds, we have compared the physicochemical properties of CC, DC’, NP and TCM-Mesh phytochemicals with IMPPAT phytochemicals (Fig. 4c). In terms of the six physicochemical properties, the IMPPAT phytochemicals are found to be more similar to TCM-Mesh phytochemicals in comparison to NP, DC’ or CC (Fig. 4c). The above results underscore the importance of our curated collection of 9596 IMPPAT phytochemicals from Indian medicinal plants which will be a valuable addition to natural product-based screening collections.

### Druggability analysis of phytochemicals of Indian medicinal plants

**Figure 5:**
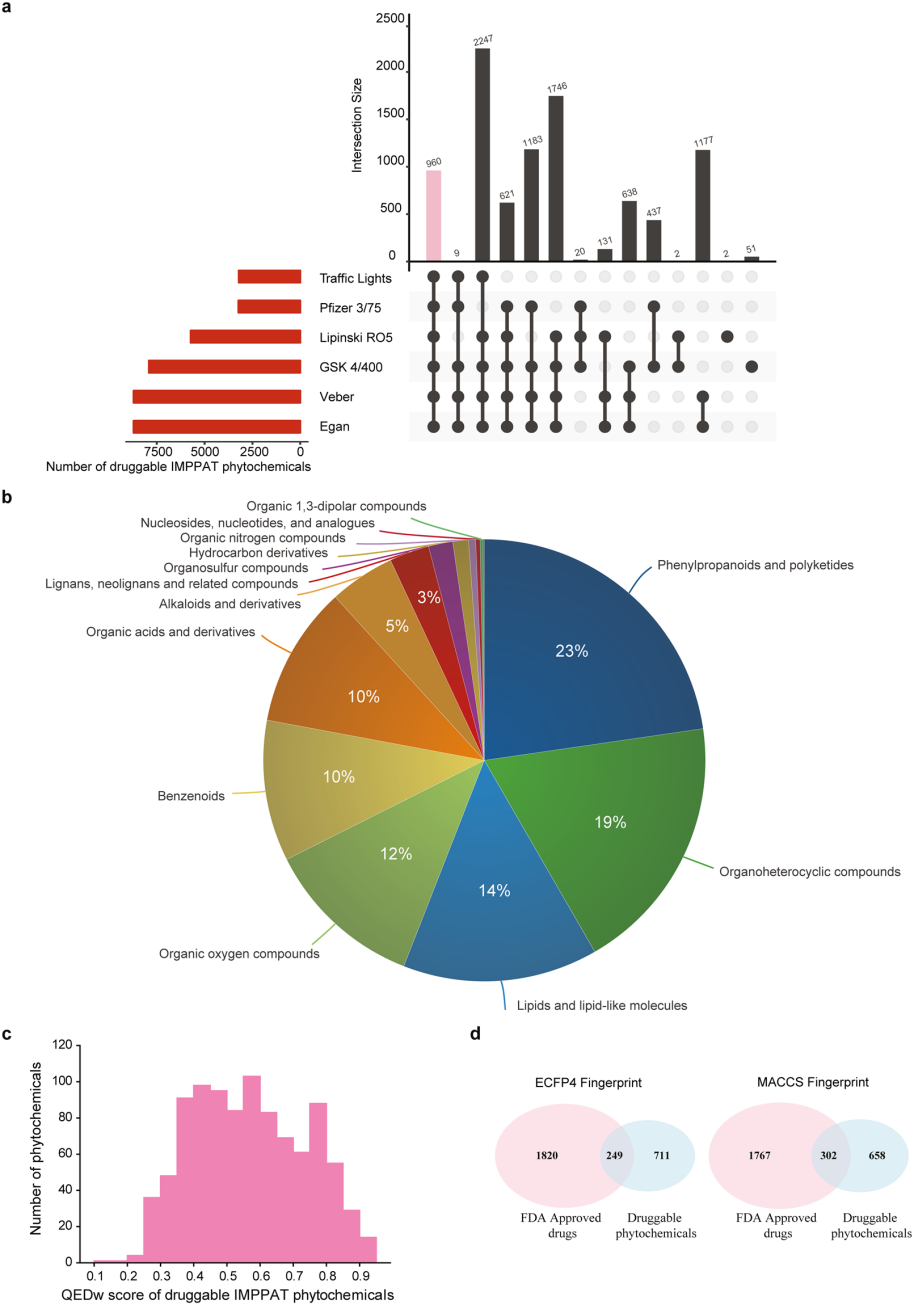
Druggability analysis of phytochemicals in IMPPAT database. (**a**) Evaluation of drug-likeliness of phytochemicals based on multiple scores. The horizontal bar plot shows the number of phytochemicals in the IMPPAT database that satisfy different druggability scores (Methods). The vertical bar plot shows the overlap between sets of phytochemicals that satisfy different druggability scores. The pink bar in the vertical plot gives the 960 phytochemicals which satisfy all druggability scores. This plot was generated using UpSetR^105^ package. (**b**) Classification of the 960 druggable phytochemicals into chemical super-classes obtained from ClassyFire^50^. (**c**) Distribution of QEDw^91^ scores for the 960 IMPPAT phytochemicals which satisfy all druggability scores. (**d**) Venn diagrams summarizing structural similarity analysis of 960 druggable phytochemicals in IMPPAT database and FDA approved drugs. Based on ECFP4 and MACCS keys molecular fingerprints, 249 and 302 druggable phytochemicals, respectively, were found to be similar to FDA approved drugs.

We evaluated the druggability of 9596 IMPPAT phytochemicals based on multiple rules or scoring schemes, namely, RO5^23^, Traffic Lights^24^, GSK’s 4/400^25^, Pfizer’s 3/75^26^, Veber rule^27^ and Egan rule^28^ which were computed using FAF-Drugs4 webserver^20^ (Methods). The horizontal bar plot in Fig. 5a gives the number of IMPPAT phytochemicals that satisfy different druggability scores. From this figure, it is seen that the majority of IMPPAT phytochemicals satisfy Veber rule or Egan rule in comparison to Pfizer’s 3/75 rule or net Traffic Lights value of zero. Furthermore, we find that the same set of 8712 IMPPAT phytochemicals satisfy both the Veber rule and Egan rule for drug-likeliness. The vertical bar plot of Fig. 5a shows the overlap between sets of phytochemicals that satisfy different druggability scores. We found that 960 out of 9596 IMPPAT phytochemicals satisfy all evaluated druggability scores (Fig. 5a). Subsequently, we designated this filtered list of 960 IMPPAT phytochemicals as *druggable*. Among the 1742 Indian medicinal plants in our database, *Brassica oleracea, Catharanthus roseus, Zea mays, Oryza sativa, Vigna radiate, Pisum sativum, Anethum sowa, Allium cepa, Cassia obtusifolia* and *Camellia sinensis* produce the highest number of druggable phytochemicals, and Supplementary Table S3 gives the number of druggable phytochemicals for each plant in IMPPAT database. In Fig. 5b, we show the distribution of the 960 druggable IMPPAT phytochemicals across different chemical super-classes obtained using ClassyFire^50^. Among the chemical super-classes, phenylpropanoids and polyketides, organoheterocyclic compounds, and lipids and lipid-like molecules are the top three with 218, 182 and 137 phytochemicals, respectively (Fig. 5b; Methods). Among the chemical classes, organooxygen compounds, prenol lipids, and flavonoids are the top three with 111, 97 and 96 phytochemicals, respectively. Moreover, organooxygen class includes many carbohydrates, carbonyl compounds and alcohols while the prenol lipids class mainly comprises different types of terpenes and terpenoids. Figure 5c shows the distribution of QEDw^91^ scores for the 960 druggable IMPPAT phytochemicals with mean score of 0.57 and standard deviation of 0.17 (Methods). From this figure, it is seen that 14 druggable phytochemicals have a QEDw score ≥0.9 and 98 druggable phytochemicals have a QEDw score ≥0.8.

By comparing the 2069 FDA approved drugs from the DrugBank^19^ with the 960 druggable IMPPAT phytochemicals, we found that only 32 FDA approved drugs are among the 960 phytochemicals while the remaining 928 phytochemicals are potential new hits. By investigating the structural similarity between 2069 FDA approved drugs and 960 druggable IMPPAT phytochemicals, we found that 249 and 302 druggable phytochemicals are similar to FDA approved drugs based on ECFP4 or MACCS keys molecular fingerprints, respectively (Fig. 5d; Methods). Combined, ECFP4 and MACCS keys based fingerprints identified 369 out of 960 druggable IMPPAT phytochemicals that are similar to FDA approved drugs (Methods). Thus, almost 40% of the druggable IMPPAT phytochemicals are similar to at least one FDA approved drug which testifies to our systemic approach to identify potential druggable phytochemicals of Indian medicinal plants. Importantly, the remaining 591 druggable IMPPAT phytochemicals which have no similarity with any of the FDA approved drugs are novel candidates for designing new drugs based on natural products from Indian medicinal plants.

For subsequent analysis, we selected 14 druggable phytochemicals with QEDw score^91^ ≥0.9 which were designated as the *most-druggable* phytochemicals. Of these 14 phytochemicals, 12 were found to be similar to at least one of the FDA approved drugs based on either ECFP4 or MACCS keys based molecular fingerprint. The remaining 2 most-druggable phytochemicals, Onosmone (CID:102212116) and Truxillic acid (CID:78213), were found to have no similarity with any of the FDA approved drugs. Supplementary Figure S1a displays the similarity matrix based on Tc obtained using ECFP4 molecular fingerprint for the 14 most-druggable phytochemicals (Methods). From the similarity matrix, it is seen that 75 of the 91 Tc values between the 14 most-druggable phytochemicals are <0.5 implying high structural diversity. Moreover, the similarity matrix can be transformed into a similarity network for the 14 most-druggable phytochemicals by using a stringent threshold value of Tc ≥0.5 to determine edges in the graph (Supplementary Figure S1b; Methods). The similarity network for the 14 most-druggable phytochemicals has 16 edges and can be partitioned into a large connected component (cluster) of 7 phytochemicals, a smaller connected component of 2 phytochemicals and 5 remaining isolated phytochemicals. We highlight that the 2 phytochemicals, Onosmone and Truxillic acid, that have no similarity with any of the FDA approved drugs are among the isolated nodes in the similarity network (Supplementary Figure S1b). Based on plant-phytochemical associations in our database, Onosmone and Truxillic acid are phytochemicals of Indian medicinal plants, *Onosma echioides* and *Erythroxylum coca*, respectively, and a survey of the literature shows that these phytochemicals are under active investigation for their therapeutic uses^99–103^. We also highlight that none of the 14 most-druggable phytochemicals are captured by Phytochemica^11^ database while 6 of the 14 phytochemicals are captured by Nutrichem^9,10^ database.

We also investigated the physicochemical properties of the 14 most-druggable phytochemicals. A principal component analysis (PCA) of the 14 most-druggable phytochemicals based on their physiochemical properties revealed that the first and second principal components together explained 69% of the total variance in the dataset (Supplementary Figure S1c). We find that some of the 7 most-druggable phytochemicals which are clustered together in the structural similarity space (Supplementary Figure S1b) are not clustered together in the physicochemical space (Supplementary Figure S1c). These observations based on limited analysis of 14 most-druggable phytochemicals suggest that a combined exploration of structural similarity space, physicochemical space and biological activity space of IMPPAT phytochemicals will facilitate identification and design of novel drugs. Thus, in future, it will be also worthwhile to compile biological activity profiles for phytochemicals of Indian medicinal plants.

### Comparison with phytochemical space of Chinese medicinal plants

**Figure 6:**
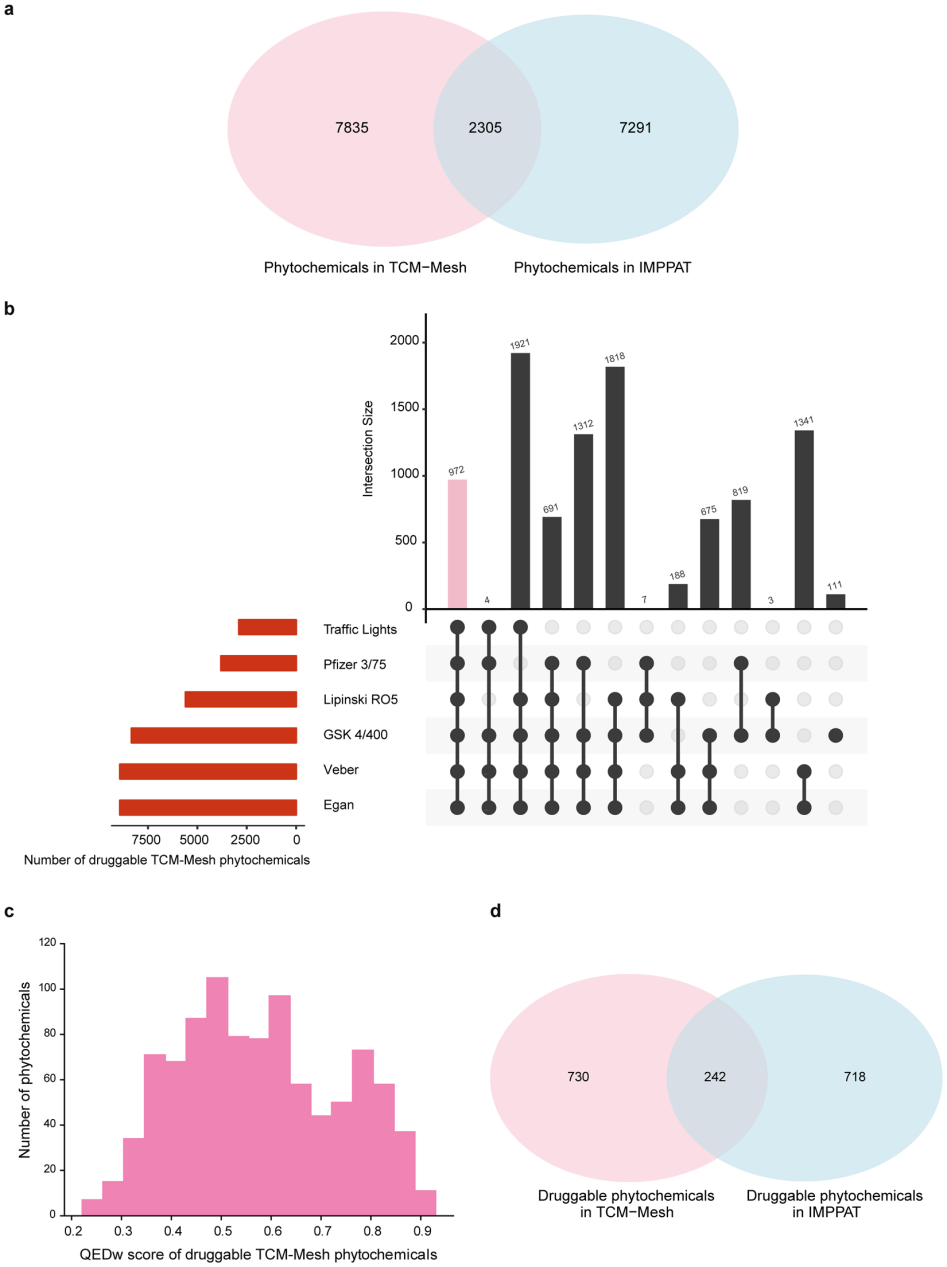
Comparison of the phytochemical space of Indian and Chinese medicinal plants. (**a**) Venn diagram shows the overlap of the phytochemicals in IMPPAT and TCM-Mesh database. (**b**) Evaluation of the drug-likeliness of TCM-Mesh phytochemicals based on multiple scores. The horizontal bar plot shows the number of phytochemicals in the TCM-Mesh database that satisfy different druggability scores (Methods). The vertical bar plot shows the overlap between sets of TCM-Mesh phytochemicals that satisfy different druggability scores. The pink bar in the vertical plot gives the 972 phytochemicals in TCM-Mesh database which satisfy all druggability scores. (**c**) Distribution of QEDw^91^ scores for the 972 TCM-Mesh phytochemicals which satisfy all druggability scores. (**d**) Venn diagram shows the overlap between the druggable phytochemicals in IMPPAT database and TCM-Mesh database.

We have compared the set of 9596 IMPPAT phytochemicals produced by Indian medicinal plants with the set of 10140 TCM-Mesh^16^ phytochemicals produced by Chinese medicinal plants (Methods). By comparing the 9596 IMPPAT phytochemicals with 10140 TCM-Mesh phytochemicals, we find that less than 25%, specifically 2305 phytochemicals, are common between the two databases (Fig. 6a). Among the 9596 IMPPAT phytochemicals, a subset of 960 phytochemicals were found to be druggable based on multiple druggability scores, namely, RO5^23^, Traffic Lights^24^, GSK’s 4/400^25^, Pfizer’s 3/75^26^, Veber rule^27^ and Egan rule^28^ (Fig. 5a). Among the 10140 TCM-Mesh phytochemicals, we found a subset of 972 phytochemicals to be druggable based on multiple druggability scores listed above (Fig. 6b). Thus, the relative size of the filtered subset of druggable phytochemicals is very similar for IMPPAT database (10%) on Indian medicinal plants and TCM-Mesh database (9.6%) on Chinese medicinal plants. Figure 6c shows the distribution of QEDw^91^ scores for the 972 druggable TCM-Mesh phytochemicals with mean score of 0.58 and standard deviation of 0.16, and thus, this distribution is similar to that for 960 druggable IMPPAT phytochemicals shown in Fig. 5c. By comparing the set of FDA approved drugs with the subset of druggable phytochemicals in IMPPAT and TCM-Mesh, we find that 32 approved drugs are contained in 960 IMPPAT phytochemicals while 19 approved drugs are contained in 972 TCM-Mesh phytochemicals. Thus, we find that majority of druggable phytochemicals in both IMPPAT (928 phytochemicals) and TCM-Mesh (953 phytochemicals) are potential hits for future drug discovery. Lastly, we find only a small overlap of 242 phytochemicals between the set of 960 druggable IMPPAT phytochemicals and 972 druggable TCM-Mesh phytochemicals (Fig. 6d), and thus, phytochemicals from both Indian herbs and Chinese herbs offer extensive opportunity for novel drug discovery.

## Discussion and future directions

Cheminformatics can accelerate drug discovery from diverse natural sources^5^. We here incorporate cheminformatic principles to build an extensive resource on phytochemistry and ethnopharmacology of Indian medicinal plants. Here we present, IMPPAT, a curated database of 1742 Indian Medicinal Plants, 9596 Phytochemicals, And 1124 T herapeutic uses which is the largest, freely accessible, digital resource on natural products from Indian herbs to date. IMPPAT provides chemical classification, 2D and 3D chemical structure, physicochemical properties, predicted ADMET properties, drug-likeliness scores and predicted human target proteins for phytochemicals in the database, and the available information in the database can be used for virtual screening. IMPPAT also captures limited information on the associations between Indian medicinal plants and their use in traditional Indian medicinal formulations. Thus, IMPPAT provides a unifying platform for the application of computational approaches to elucidate mechanistic links between phytochemicals of Indian medicinal plants and their therapeutic action.

Following Clemons *et al*.^78^, we have compared the distributions of stereochemical complexity and shape complexity (Fsp^3^) across 9596 IMPPAT phytochemicals with small molecule collections, CC, DC’ and NP and 10140 TCM-Mesh^16^ phytochemicals from Chinese medicinal plants (Fig. 4a–b). Interestingly, we show that the mean and median of stereochemical complexity or shape complexity of IMPPAT phytochemicals is closer to NP or TCM-Mesh phytochemicals than CC or DC’ collections. Following Clemons *et al*.^98^, we have also compared six physicochemical properties of CC, DC’, NP and TCM-Mesh phytochemicals with IMPPAT phytochemicals (Fig. 4c) to show that IMPPAT phytochemicals are closer to TCM-Mesh phytochemicals in physicochemical space (Fig. 4c). These results suggest that the IMPPAT library of phytochemicals is more likely to be enriched for specific protein binders rather than promiscuous binders^78^, and thus, our phytochemical library is expected to be a valuable addition to natural product-based screening collections.

Using cheminformatic approaches, we found that 960 of the 9596 IMPPAT phytochemicals of Indian medicinal plants are potentially druggable based on multiple scoring schemes. Of the 960 IMPPAT phytochemicals which satisfy all druggability scores evaluated here, a subset of 14 phytochemicals were found to have a QEDw^91^ score ≥0.9 (Supplementary Figure S1). Interestingly, the occurrence of these 14 most-druggable phytochemicals across 1742 Indian medicinal plants in our database is very rare with none of the 14 phytochemicals being found in more than 3 Indian medicinal plants. Specifically, the 14 most-druggable phytochemicals are constituents of only 17 Indian medicinal plants in our database. Also, 4 of the 14 most-druggable phytochemicals are constituents of 3 phylogenetically close Indian medicinal plants, *Iris germanica, Iris nepalensis* and *Iris kemaonensis*, which are from the same genus. However, we find that only 2 out of 17 Indian medicinal plants that produce the 14 most-druggable phytochemicals are in the priority list of Ministry of AYUSH, Government of India. This analysis suggests a possible revision in the AYUSH priority list to include the remaining 15 Indian medicinal plants that produced the majority of the most-druggable phytochemicals in our database. Thus, our resource will also aid in future expansion of the chemotaxonomy^104^ of Indian medicinal plants.

Lastly, we have also compared the IMPPAT phytochemicals from Indian herbs with the TCM-Mesh^16^ phytochemicals from Chinese herbs to show that roughly 75% of the phytochemicals are unique to each database (Fig. 6). Moreover, we found that the filtered subsets of druggable phytochemicals in IMPPAT and TCM-Mesh are similar in size with roughly 75% of druggable phytochemicals unique to each database (Fig. 6). Furthermore, among the 960 and 972 druggable phytochemicals in IMPPAT and TCM-Mesh, respectively, a small fraction of 32 and 19 approved FDA drugs are contained in IMPPAT and TCM-Mesh, respectively. In sum, our results underline the vast potential of both Indian and Chinese herbs for future drug discovery.

In the future, we hope to update IMPPAT database with the following additional information. Firstly, it will be important to update our database with more detailed information on the parts of the Indian medicinal plants such as leaves, stem or root, that produce the different phytochemicals along with relative composition of phytochemicals in different parts of the plants. Such detailed information on the relative phytochemical composition of parts of Indian medicinal plants will be crucial for evaluating and developing traditional Indian medicine formulations^74^. However, significant manual curation and literature mining will be needed to expand our database to include the relative phytochemical composition of the different parts of 1742 Indian medicinal plants which is beyond the scope of the present work. Secondly, it will be important to enrich our database by incorporating more traditional Indian medicinal formulations. For example, TKDL (http://www.tkdl.res.in) has made only 1200 of their documented 250000 traditional Indian medicinal formulations openly accessible, and future efforts to associate this wealth of information to our database will shed mechanistic information on the therapeutic action of traditional formulations. Thirdly, it will be important to compile known biological activity information for phytochemicals of Indian medicinal plants. In conclusion, IMPPAT database will serve as a valuable resource in herbal drug discovery.

## Electronic supplementary material

Supplementary Figure 1

Supplementary Tables S1-S3
