# Supplementary material for: IMPPAT: A curated database of Indian Medicinal Plants, Phytochemistry And Therapeutics
Source: Sci Rep. 2018 Mar 12;8:4329. doi: 10.1038/s41598-018-22631-z (PMC5847565; doi:10.1038/s41598-018-22631-z)
Supplement: Supplementary file 1 — Supplementary Figure 1 [file 41598_2018_22631_MOESM1_ESM.docx]

**Supplementary Figure S1**

**of**

IMPPAT: A curated database of Indian Medicinal Plants, Phytochemistry And Therapeutics

**Karthikeyan Mohanraj^1,#^, Bagavathy Shanmugam Karthikeyan^1,#^, R.P. Vivek-Ananth^1,#^, R.P. Bharath Chand^1^, S.R. Aparna^2^, P. Mangalapandi^1^, Areejit Samal^1,^**^*^

^1^The Institute of Mathematical Sciences (IMSc), Homi Bhabha National Institute, Chennai 600113, India

^2^Stella Maris College, Chennai 600086, India

^#^K.M, B.S.K. and R.P.V. contributed equally to this work

^*^Corresponding author: [asamal@imsc.res.in](mailto:asamal@imsc.res.in)

**
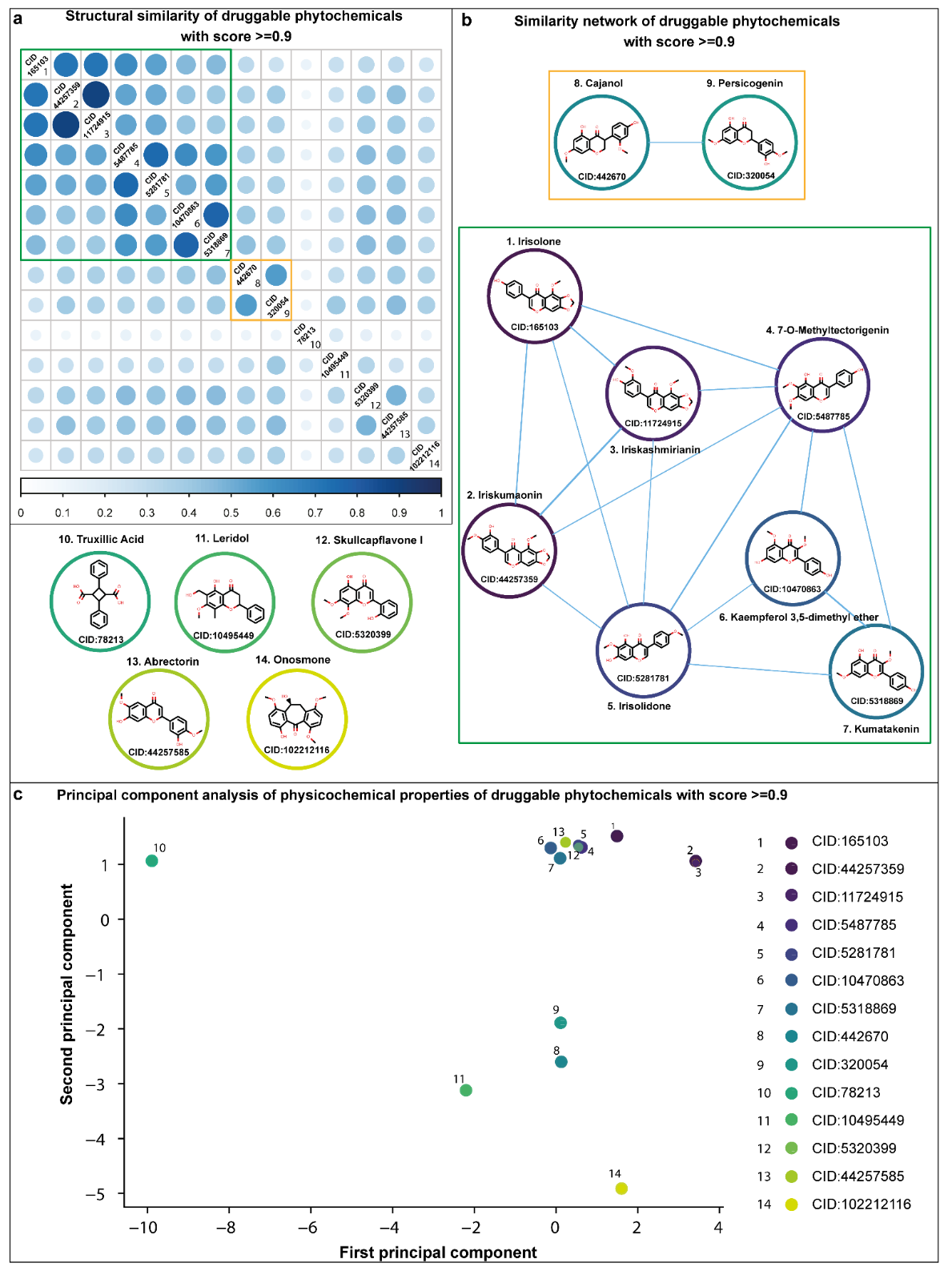
**

**Supplementary Figure S1:** **Structural similarity and physicochemical properties of most-druggable phytochemicals in IMPPAT database.** (a) Similarity matrix for the 14 most-druggable phytochemicals with QEDw score ≥ 0.9 based on Tanimoto coefficient (Tc) between pairs of chemicals computed using ECFP4 molecular fingerprints. Note the chemical identifiers of 14 most-druggable phytochemicals in the similarity matrix have been consistently numbered from 1 to 14 across different panels of this figure. (b) Similarity network for the 14 most-druggable phytochemicals constructed using a stringent threshold value of Tc ≥ 0.5 to determine edges in the graph. We find that the similarity network can be partitioned into a large connected component of 7 phytochemicals, a smaller connected component of 2 phytochemicals and 5 isolated phytochemicals. (c) Principal component analysis (PCA) of the 14 most-druggable phytochemicals based on their physicochemical properties. The first and second principal components can together explain 69% of the total variance in the dataset.
